# Review of Pediatric Pheochromocytoma and Paraganglioma

**DOI:** 10.3389/fped.2017.00155

**Published:** 2017-07-13

**Authors:** Reshma Bholah, Timothy Edward Bunchman

**Affiliations:** ^1^Pediatric Nephrology, Virginia Commonwealth University, Richmond, VA, United States

**Keywords:** pheochromocytoma, paraganglioma, pediatrics, SDHx hereditary paraganglioma–pheochromocytoma syndromes, phenoxybenzamine, metyrosine, long-term follow-up

## Abstract

Pheochromocytoma (PCC) and paraganglioma (PGL) are rare chromaffin cell tumors which secrete catecholamines and form part of the family of neuroendocrine tumors. Although a rare cause of secondary hypertension in pediatrics, the presentation of hypertension in these patients is characteristic, and treatment is definitive. The gold standard for diagnosis is *via* measurement of plasma free metanephrines, with imaging studies performed for localization, identification of metastatic lesions and for surgical resection. Preoperative therapy with alpha-blocking agents, beta blockers, and potentially tyrosine hydroxylase inhibitors aid in a safe pre-, intra- and postoperative course. PCC and PGL are inherited in as much as 80% of pediatric cases, and all patients with mutations should be followed closely given the risk of recurrence and malignancy. While the presentation of chromaffin cell tumors has been well described with multiple endocrine neoplasia, NF1, and Von Hippel–Lindau syndromes, the identification of new gene mutations leading to chromaffin cell tumors at a young age is changing the landscape of how clinicians approach such cases. The paraganglioma–pheochromocytoma syndromes (SDHx) comprise familial gene mutations, of which the SDHB gene mutation carries a high rate of malignancy. Since the inheritance rate of such tumors is higher than previously described, genetic screening is recommended in all patients, and lifelong follow-up for recurrent tumors is a must. A multidisciplinary team approach allows for optimal health-care delivery in such children. This review serves to provide an overview of pediatric PCC and PGL, including updates on the preferred methods of imaging, guidelines on gene testing as well as management of hypertension in such patients.

## Introduction

The rare neuroendocrine tumors pheochromocytoma (PCC) and paraganglioma (PGL) are the cause of hypertension in 0.5–2% of pediatric cases (1, 2). PCCs arise from the adrenal medulla and comprise 80–85% of catecholamine-secreting tumors while PGLs arise from extra-adrenal locations and are subdivided into sympathetic and parasympathetic PGLs, accounting for 15–20% of these tumors (3). Sympathetic PGLs arise along the sympathetic ganglion chain (4) in the chest, abdomen, and pelvis. Parasympathetic PGLs arise from parasympathetic tissue in the head and neck (HNPGL); these rarely secrete catecholamines. PCCs and PGLs have different catecholamine-secreting profiles. Tyrosine is the precursor to catecholamines, which through a series of enzymatic reactions is converted to DOPA by the enzyme tyrosine hydroxylase. DOPA is converted to dopamine, which is further converted to norepinephrine and finally changed to epinephrine. The distinction between the types of hormones secreted by adrenal or extra-adrenal tumors comes from the enzyme phenylethanolamine *N*-methyltransferase (PNMT) present in the adrenal gland, with its expression dependent upon onsite cortisol. PNMT can convert norepinephrine to epinephrine (5) and as such, tumors secreting epinephrine and frequently norepinephrine are generally from the adrenal gland while extra-adrenal tumors secrete norepinephrine and dopamine. PCCs and PGLs occur sporadically as well as in the context of hereditary syndromes to include multiple endocrine neoplasia (MEN) type 2, Von Hippel–Lindau (VHL) type 2, neurofibromatosis (NF) type 1, and the paraganglioma–pheochromocytoma syndromes (SDHx). The reported inheritance has changed from 30 to 40% in small pediatric case series (1, 6–10) to 80% in a larger series (11) and other susceptibility genes, not currently ascribed to syndromes, have been identified.

## Causes of Hypertension in Pediatrics

The overall prevalence of hypertension has risen from 2 to 4.5% (12, 13) in the pediatric population, with much of this increase attributed to obesity induced hypertension. Secondary hypertension is more common in younger children resulting from reno-vascular or renal parenchymal disease (78–80% of causes) (14, 15), endocrine (11% of causes) (14), cardiac (2% of causes) (16), pulmonary and others as shown in Table 1. Since PCCs and PGLs account for only 0.5–2% (2) of secondary hypertension, one should be mindful to rule out more common causes when evaluating a child with elevated blood pressure (BP). As depicted in Table 1, taking into account symptomatology, as well as laboratory findings and a family history may point toward a catecholamine-secreting tumor.

**Table 1:** Secondary causes of hypertension by organ system with clinical and laboratory findings.

Organ system	Differential diagnosis	Findings/workup

Renal		
Renal parenchyma	Acute and chronic glomerulonephritis	Hematuria, proteinuria, edema
	Acute and chronic renal failure	Use KDIGO, pRIFLE, or AKIN guidelines for diagnosis
	Congenital renal malformations^a^	Prenatal/postnatal renal US findings of dysplasia, obstructive uropathy
	Polycystic kidney disease	Hepatosplenomegaly (ARPKD)
	Systemic vasculitis	
	SLE	Low C3, C4, CH50, +ds-DNA, +anti-Smith, joint pain/swelling, rash, edema
	ANCA	Normal to +ANCA, ↑ CRP, ↑ ESR, joint pain/swelling, rash, edema
	HSP	Hematuria, proteinuria, purpuric rash
	PAN	Arteriography, ↑ liver enzymes, livedo reticularis
	Parenchymal scar from pyelonephritis, VUR, HUS	DMSA scan; VCUG and history of UTIs; hemolysis, uremia, +/− diarrhea, AKI

Reno-vascular	Renal vein thrombosis^a^	Hematuria, thrombocytopenia, flank mass
	Renal artery stenosis	Abdominal bruit, angiogram, and renal vein sampling
	Fibromuscular dysplasia	
	Syndromes	
	Williams	Elfin facies, short stature, hypercalcemia, supravalvular aortic stenosis, “cocktail party” personality, CAKUT
	Turners	Webbed neck, widely spaced nipples, short stature, ovarian failure, cardiac malformation, CAKUT
	NF1	Neurofibromas, café-au-lait spots, axillary freckling, Lisch nodules, optic gliomas, bone and CNS abnormalities
	Arteritis	
	Takayasu’s	Bruit, angiogram
	Kawasaki Moyamoya	Conjunctival injection, strawberry tongue, erythema of the extremities, cervical lymphadenopathy, polymorphous rash, ↑ WBCs and platelets, ↑ liver enzymes, ↑ ESR, ↑ CRP TIA, stroke, epilepsy, EEG, head CT/MRI, angiogram
	Renal transplant artery stenosis	Bruit, angiogram
	Tumors compressing on renal vessels	Angiogram

Endocrine	Catecholamine excess	
	Pheochromocytoma/paraganglioma	Flushing, diaphoresis, tachycardia, abdominal mass
	Neuroblastoma Sympathomimetic drugs: phenylpropanolamine (decongestant), cocaine, amphetamine, phencyclidine, epinephrine, phenylephrine, terbutaline, monoamine oxidase-inhibitor with tyramine containing foods	Tachycardia, abdominal mass, CT/MRI, ↑ urine and serum catecholamines, biopsy
	Corticosteroid excess	
	Cushing syndrome:	
	ACTH dependent	
	ACTH independent	
	Mineralocorticoid excess	Truncal obesity, moon facies, abdominal striae, hirsutism
	Congenital adrenal hyperplasia	↑ ACTH; brain MRI
	Aldosterone-secreting tumors	↓ ACTH; CT/MRI abdomen
	Thyroid disease Hyperthyroidism Hypothyroidism	Ambiguous genitalia/virilization (girls), phallic enlargement/scrotal hyperpigmentation (boys); ↑ 17-hydroxyprogesterone (21-hydroxylase deficiency); hyponatremia, hyperkalemia, FTT (boys) ↑ Aldosterone, ↓ PRA, hypokalemia, metabolic alkalosis Nervousness, exophthalmos (Graves’ disease), muscle tremors, weight loss, heat intolerance, thinning skin/fine hair, frequent bowel movements; ↓ TSH, ↑ T4
	Hypercalcemia (primary or secondary to malignancy, hyperparathyroidism, vitamin D intoxication)	Fatigue, muscle cramps/weakness, weight gain, dry/coarse skin and thinning hair, cold intolerance, constipation; ↑TSH, ↓T4

Cardiac	Coarctation of the aorta^a^ Mid aortic syndrome^a^	Radio-femoral delay of pulses, normal/low blood pressure in legs, heart murmur

Pulmonary	Obstructive sleep apnea	Snoring
	Bronchopulmonary dysplasia^a^	Supplemental oxygen requirement for >28 days in neonates (see ATS diagnostic criteria)

Central nervous system	Elevated intracranial pressure Seizures	Bradycardia

Medications	Steroids	Moon facies, abdominal striae
	Immunosuppressants	
	Cyclosporine	Hypertriglyceridemia, hypertrichosis, gingival hyperplasia, hirsutism, headache, tremors, aphthous ulcers
	Tacrolimus	Hyperkalemia, hypomagnesemia, tremors, hyperglycemia
	Sirolimus	Impaired wound healing, dyslipidemia, myopathy, liver dysfunction
	Oral contraceptives Anesthetics: ketamine Erythropoietin	

Monogenic HTN	Liddle’s syndrome	Hypokalemia, metabolic alkalosis, low PRA and aldosterone
	Gordon’s syndrome (pseudohypoaldosteronism type II)	Hyperkalemia, low/low normal PRA and aldosterone
	Syndrome of apparent mineralocorticoid excess	Hypokalemia, metabolic alkalosis, low PRA and aldosterone, FTT, elevated ratio of urinary tetrahydrocortisol + allotetrahydrocortisol/tetrahydrocortisone, hypercalciuria
	Glucocorticoid remediable aldosteronism (aka familial hyperaldosteronism type I)	Hypokalemia, metabolic alkalosis, normal/high urinary aldosterone, 18-oxo-tetrahydrocortisol/tetrahydrocortisol >1

Miscellaneous	Post ECMO^a^	
	Cyclical vomiting syndrome	Vomiting, hyponatremia, migraines

*Adapted from Kapur and Baracco (16) and Brady and Feld (14)*.

*^a^Common etiology of HTN in neonates and infants*.

*ACTH, adenocorticotrophic hormone; ANCA, antineutrophil cytoplasmic antibody; ATS, American thoracic society; CAKUT, congenital anomalies of the kidney and urinary tract; CRP, C-reactive protein; CT, computed tomography; ECMO, extra-corporeal membrane oxygenation; ESR, erythrocyte sedimentation rate; FTT, failure to thrive; HSP, henoch schonlein purpura; HUS, hemolytic uremic syndrome; MRI, magnetic resonance imaging; PAN, polyarteritis nodosa; PRA, plasma renin activity; SLE, systemic lupus erythematosus; TIA, transient ischemic attack; VUR, vesico-ureteral reflux; WBC, white blood cell*.

## Clinical Presentation

The average age at presentation of PCCs and PGLs in pediatrics is 11–13 years, with a male preponderance of 2:1 (11, 17, 18). The clinical presentation is variable, with sustained hypertension seen in 60–90% of pediatric cases (17–19). In contrast, adults exhibit paroxysmal hypertension in about 50% of cases. One case series described 67% of children with headaches in addition to hypertension (17), while palpitations, sweating, pallor, nausea, and flushing were seen in 47–57% of children (1, 3, 4, 17, 19, 20). Anxiety, weight loss, and visual disturbance (7) were manifested in some while polyuria and polydipsia were reported as the only presenting symptoms in one case study (21). Symptomatology may depend on the type of hormone being secreted. Individuals with epinephrine secreting tumors can present with hypoglycemia and hypotensive shock, from excess catecholamine production and circulatory collapse. Dopamine-secreting tumors are usually asymptomatic, delaying diagnosis until the mass effect of the tumor is apparent (22). The mass effect from non-functional head and neck paragangliomas (HNPGLs) can lead to dysphagia, hoarseness, hearing disturbances, and pain.

## Genetics of PCC and PGL

Contrary to historic belief of the “rule of 10” for PCCs and PGLs that 10% are hereditary, 10% are malignant, 10% are extra-adrenal, and 10% are bilateral, their inheritance is much higher in reported pediatric case series. The European-American-Pheochromocytoma-Paraganglioma-Registry (EAPPR) followed 164 unrelated pediatric patients diagnosed with PCCs/PGLs, 80% of which had a germline mutation in a gene associated with such tumors (11). Previous to this registry, the estimated percentage of germline mutations was 30–40% in smaller pediatric case series (1, 4, 7–10, 23). This change in prevalence is multifactorial and may result from increased screenings for mutations, novel identification of mutations as well as data from the EAPPR being more reflective of a population-based frequency.

In addition to the known syndromic presentations of MEN II, NF1, and VHL, germline succinate dehydrogenase gene mutations (SDHx) involving the mitochondrial enzyme complex (SDH) form part of the familial PGL–PCC syndromes. SDH consists of the four subunits SDHA, SDHB, SDHC, and SDHD; SDHAF2 is one of the factors involved in the assembly of the SDH complex. These syndromes are inherited in an autosomal dominant fashion with varying penetrance. The Carney triad syndrome (described in 1977), Carney–Stratakis syndrome (described in 2002) and Pacak–Zhuang syndrome (described in 2013) are rare syndromes with PGLs as one of the presenting features.

The Carney triad syndrome constitutes gastrointestinal stromal tumors (GISTs), PGLs, and pulmonary chondromas. There is no known genetic defect identified to date. Sporadic GISTs are distinct from those associated with Carney triad in the staining pattern they exhibit. In pediatrics, GISTs are found in the stomach with negative SDHB staining, which is defined as a loss of a granular cytoplasmic pattern in the presence of valid positive controls (24). In adults, GIST is primarily found along the GI tract excluding the stomach, and these tumors are SDHB positive. When adult patients have GIST originating from the stomach, the histology and staining of these tumors resemble pediatric GIST (24). The mean age of presentation is 21 years with young women being predominantly affected. In one study of 79 patients, 47% presented with PGLs or PCCs, the majority of which (37 patients) were PGLs (92%) (25, 26).

The Carney–Stratakis syndrome has an autosomal dominant pattern of inheritance and is a diad that comprises of GISTs and PGLs. Most patients have been found to carry germline mutations in SDHB, SDHC or SDHD. It is rare and was identified in about 20 kindreds (27); 58% of 12 patients with this syndrome only had PCC/PGLs with the youngest child reported in the literature being 12 years old (28).

The Pacak–Zhuang syndrome has the gene mutation in hypoxia-inducible factor 2 alpha with clinical features of HNPGLs, somatostatinomas, and polycythemia (29). In a series of seven patients, of which three were pediatric cases with PCCs and/or PGLs, all patients presented with polycythemia at birth or in early childhood (30). The earliest age of presentation with PCC/PGL was 8, with a median age of 17. Somatostatinomas developed in 5 of the 7 patients at a median age of 29 (range 22–38), and erythropoietin levels were about fivefolds above the upper limit of normal (ULN). Common ocular complications associated with this syndrome include dilated capillaries and fibrosis overlying the optic disk (31).

Multiple other gene mutations associated with hereditary PCCs and PGLs have been identified in the past decade and include TMEM127 involved in the mTOR pathway, MAX that controls gene transcription (32, 33) as well as KIF 1B, EGLN1, IDH1, and FH, with unclear clinical significance (34). Table 2 lists the syndromic as well as newer gene mutations associated with PCCs and PGLs and describes the biochemical profile of such tumors, including the earliest age of presentation as noted in the literature.

**Table 2:** Clinical features of syndromes associated with pheochromocytoma (PCC) and paragangliomas (PGLs), as well as earliest age of diagnosis, malignancy rate, and additional information including hormone-secreting profile of tumors.

Syndromes	Gene	Clinical features	Earliest age of diagnosis (year)^c^	Malignancy rate (%)^d^	Additional information
Multiple endocrine neoplasia type 2	RET			2.9	
Type 2a		Medullary thyroid carcinoma PCC	5–8		PCCs are the first clinical manifestation in 10–30% of patients
		Hyperparathyroidism			
		Cutaneous lichen amyloidosis			
Type 2b		Medullary thyroid carcinoma	12		Penetrance of ~50%
		PCC			Produce both epinephrine and norepinephrine
		Multiple neuromas			
		Marfanoid habitus			Bilateral in 50–80% of patients (3)
FMTC		Familial medullary thyroid carcinoma			

Von Hippel–Lindau syndrome type 2	VHL		5	3 (11)	
Type 2a		Retinal and CNS hemangioblastomas			PCCs present in 10–20% of patients in adult series (3) versus 6–49% of pediatric cases (6, 9, 11, 17, 36)
		PCC (often bilateral)			
		Endolymphatic sac tumors			
		Epididymal cystadenomas			
Type 2b		Renal-cell cysts and carcinomas			Produce norepinephrine
		Retinal and CNS hemangioblastomas			
		Pancreatic neoplasms and cysts			
		PCC (often bilateral)			
		Endolymphatic sac tumors			
		Epididymis cystadenomas			
Type 2c		PCC (often bilateral)			

Neurofibromatosis type 1	NF1	Neurofibromas	7	9.3–33 (11)	PCCs present in 4% (11)
		Café-au-lait spots			
		PCC			Produce epinephrine and norepinephrine
		Lisch nodules			
		Optic pathway/CNS gliomas			
		GIST			

**Paraganglioma–pheochromocytoma syndromes (SDHx)**					

	SDHA	Extra-adrenal paragangliomas (PGLs) (34)	8 (35)	0–14.3	
PGL4	SDHB	HNPGL^a^	6	17–30.7 (11)	SDHB mutation in 12.5–20% (11, 17, 36) pediatric cases
		PCC^b^			
		Extra-adrenal PGLs			
		GIST			SDHD mutation in 10% pediatric PCC cases (11)
		Renal cell carcinoma			
PGL3	SDHC	HNPGL^a^	12	–	Produce norepinephrine and rarely dopamine
		GIST			
PGL1	SDHD	HNPGL^a^	5	3.5	
		PCC^b^			
		Extra-adrenal PGLs			
		GIST			
		Papillary thyroid carcinoma (rarely)			
PGL2	SDHAF2	HNPGL	15	–	

Pacak–Zhuang syndrome (29)	HIF2A	HNPGL	11–17 (30, 37)	–	Described in 4 pediatric patients
		Somastostatinoma			
		Polycythemia			

Syndrome not described	TMEM 127 (32)	PCC		4.3–12 (35)	No pediatric case reports
		HNPGL			
		Extra-adrenal PGLs			

Syndrome not described	MAX (33)	PCC (often bilateral)	17 (33)	9–25 (35)	Described in 1 pediatric patient

*Adapted from Lenders et al. (3), Havekes et al. (1, 3), and Neumann and Eng (38)*.

*CNS, central nervous system; HNPGL, head and neck paragangliomas; GIST, gastrointestinal stromal tumors; SDH, succinate dehydrogenase*.

*^a^More frequent in SDHD with a lifetime prevalence of ~90%*.

*^b^More frequent in SDHB with a prevalence of ~80% (data mostly derived from adult observations) (1)*.

*^c^Adapted from Bausch et al. (11) and Waguespack et al. (39), unless otherwise specified*.

*^d^Adapted from Welander et al. (26) unless otherwise or additionally specified*.

Advances in molecular diagnostics have identified a distinction amongst hereditary PCCs and PGLs in the two different pathways that these tumors can fall under. The pseudohypoxic response usually stabilizes hypoxia-inducible factors (HIFs) under normoxic conditions. Gene mutations in VHL, SDHx, and HIF2 confer a reduced oxidative response with angiogenesis and hypoxia and encompass cluster 1 tumors. Conversely, kinase signaling in cells is usually kept at bay by various mechanisms as these promote cell proliferation, growth and survival dysregulation. Gene mutations in RET, NF1, KIF 1B, TMEM 127, and MAX were found to activate kinase signaling pathways leading to tumors with such features, termed cluster 2 tumors. In contrast, sporadic PCC/PGLs have features from both clusters (26). A 2017 retrospective study comparing pediatric and adult hereditary PCCs and PGLs demonstrated that cluster 1 tumors were more prevalent in the pediatric population by 76 versus 39% (40).

Malignancy rates vary and are estimated to be between 12 and 47% based on small case series (4, 6, 8, 41). In contrast, 10% of pediatric patients in the EAPPR had malignant tumors, none of which were of sporadic occurrence. Those with SDHB mutations had the highest prevalence for malignancy with extra-adrenal and thoracic paraganglial tumors posing additional risk factors (11, 42). Adult studies have also shown that SDHB mutation carry a higher risk for malignancy at 13–23% (43). The European-American-Asian Pheochromocytoma-Paraganglioma Registry prospectively followed up on predominantly adult patients with the newer gene mutations SDHA, TMEM127, MAX, and SDHAF2 and determined that 12% (4/34) of SDHA mutation carriers and 10% (3/29) of TMEM127 mutation carriers had malignant tumors (35). In contrast, of the small number of MAX mutation carriers, 9% (1/11) had a malignant tumor, with the only SDHAF2 mutation carrier being devoid of malignant disease. With regards to risk for malignancy, a single center retrospective review identified tumor size >6 cm in diameter, having a PGL or having a sporadic tumor as characteristics posing an increased risk for malignancy in a logistic analysis (4).

Twelve to sixty percent of tumors are extra-adrenal in location based on small case series (4, 6, 8, 17, 19) while the EAPPR had a rate of 30%, which may be a better reflection given the large amount of registrants. Bilateral adrenal tumors are reported to be present in 24–40% of pediatric cases (6, 8, 9, 11).

### Algorithm for Genetic Testing

**Figure 1:**
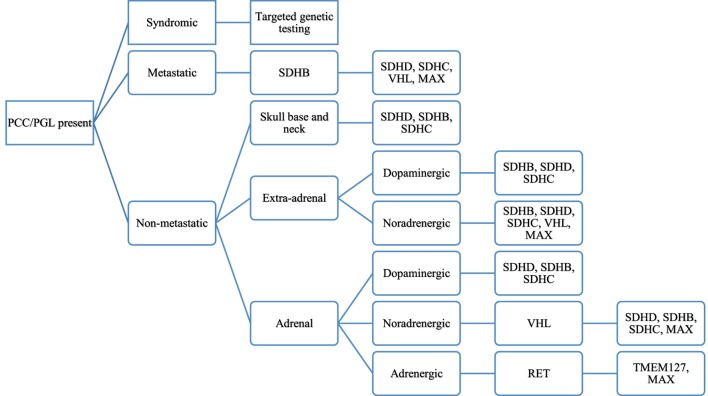
Proposed algorithm for genetic testing of patients with pheochromocytoma (PCC) or paraganglioma (PGL) based on patient’s clinical characteristics, biochemical phenotype, and clinical aspects of the tumor. Reproduced with permission from Lenders et al. (45).

While in the past one would consider the syndromes MEN2, VHL, NF1 or sporadic mutations as the cause of PCCs or PGLs, the discovery of new gene mutations that cause such tumors now needs to be incorporated into the diagnostic algorithm of patients thought to have a PCC or a PGL. An understanding of what constitutes a positive from negative staining of the SDHx genes is crucial to guide the clinician in the type of genetic testing to be performed. The SDHx subunits are found in mitochondria and exhibit a granular cytoplasmic pattern of staining if no mutation is present. Gill et al. demonstrated that SDHD mutations exhibit a weak diffuse staining pattern while SDHB mutations had a completely absent staining (44). Thus, a positive staining would entail intact SDHx subunits while a negative/weakly positive staining would indicate a mutation in the SDHx subunits (44). As can then be expected, syndromes that do not have a SDHx gene mutation, to include MEN II, VHL, and NF1, will stain positive for SDHx. Figure 1 depicts a proposed algorithm for genetic testing on patients with hereditary PCC or PGLs.

## Workup

### Laboratory Testing

**Figure 2:**
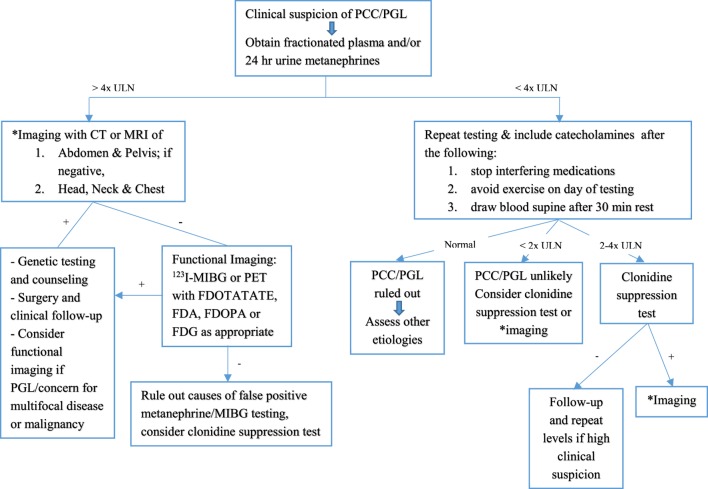
Flowchart for diagnostic algorithm for pheochromocytomas (PCCs) and paragangliomas (PGLs). +, positive; −, negative; ULN, upper limit of normal. Adapted from Waguespack et al. (39) and Dobri et al. (5).

Laboratory testing should be undertaken once there is clinical suspicion for a PCC or PGL. Measurements of plasma and 24 h urinary catecholamines (epinephrine, norepinephrine, and dopamine) and urinary vanillylmandelic acid (VMA) have fallen out of favor due to lower sensitivity and specificity (Table 3), and assessing catecholamine metabolites is now recommended. These include plasma free metanephrines (metanephrine and normetanephrine) and 24 h urinary fractionated metanephrines (46). Metabolic processes unrelated to the tumor produce VMA, decreasing its sensitivity for diagnosis. Conversely, the production of plasma free metanephrines is constant and independent of the release of catecholamines, which is episodically secreted (47), making these the gold standard for diagnosis (46, 48). The degree of elevation of catecholamine metabolites (49) ought to be assessed when evaluating for catecholamine-secreting tumors, as shown in Figure 2. Values >4 times the ULN are highly suggestive of a tumor (49) while values >ULN and <4 ULN need to be investigated further as shown in Figure 2. One needs to take into account potential confounders, listed in Table 4, which may lead to false positive and false negative results of metanephrine testing. In cases of slight elevations in plasma catecholamine metabolites, the clonidine suppression test has been used in adults to help diagnose a neuroendocrine tumor, where suppression of ≥40% of plasma metanephrines signifies the absence of a tumor (45, 49).

**Table 3:** Sensitivity and specificity of biochemical tests used in the diagnosis of pediatric pheochromocytoma.

Biochemical test	Sensitivity (%)	Specificity (%)
Plasma normetanephrine and metanephrine	100	94
Plasma norepinephrine and epinephrine	92	91
Urinary normetanephrine and metanephrine	100	95
Urinary norepinephrine and epinephrine	100	83
Urinary vanillylmandelic acid	63–75^a^	94^a^

*Adapted from Weise et al. (48), Ludwig et al. (17), and Pacak et al. (50)*.

*^a^Sensitivity of 75% was from a pediatric series (17); sensitivity of 63% and specificity of 94% were from adult data (50)*.

**Table 4:** Factors associated with false positive and false negative testing of metanephrines.

False positives
Medications (3)
Calcium channel blockers
Beta blockers
Mood stabilizers: tricyclic antidepressant, buspirone
Sympathomimetics: amphetamine, ephedrine
Stimulants: caffeine, nicotine
Dopaminergic agents: levodopa, alpha-methyldopa
Acetaminophen
Age
Increase in plasma metanephrines with age (51)
Posture
Increase in plasma metanephrines in seated versus supine position (52, 53)
Exercise (52)
High catecholamine diet (54)
Hypertension (3)
Obstructive sleep apnea (53, 55)
Stroke (3)
Renal impairment (56)

**False negatives**

Small tumors, usually <2 cm in size in normotensive patients being screened initially or for recurrence
Dopamine-secreting tumors

Tumors can rarely secrete a predominance of dopamine; these are usually extra-adrenal PGLs with SDHx gene mutations (57). Since there is no definitive constellation of symptoms pointing to dopamine-secreting tumors, their detection depends on the measurement of the dopamine metabolites methoxytyramine and homovanillic acid (22). The utility of plasma methoxytyramine as a biomarker for metastatic PCCs and PGLs was described in 2012, where patients with metastatic tumors had a 4.7-fold higher plasma methoxytyramine level than those without metastatic tumors (58).

Chromogranin A (CgA), a protein present in chromaffin cells which controls secretion of hormones from secretory granules, may improve sensitivity of diagnosing SDHB and SDHD related tumors with concomitant use of plasma metanephrines (59). Sensitivities and specificities of CgA and plasma metanephrines were 73.2/95.9 and 70.7/98.6%, respectively, in SDHB mutation carriers with PCCs and sympathetic PGLs. Sensitivity of detecting such tumors was increased by 22% (from 70.7 to 92.7%) when CgA was used in conjunction with plasma normetanephrines in these SDHB-related tumors. With regards to metastatic disease within this cohort of patients, the use of CgA in addition to plasma metanephrines improved the sensitivity of diagnosis from 75 to 94.4%. CgA levels were not a sensitive marker for diagnosing HNPGLs, where the additive effect of CgA with plasma metanephrines provided a small increase in sensitivity of diagnosis of SDHD related tumors from 71.4% for plasma metanephrines alone to 78.6% for both biomarkers (59). However, this study was limited by a small number of patients with HNPGLs, with further conclusions to be determined after investigating this biomarker on a larger group of patients with HNPGLs. The results of this study suggest that initial elevations of CgA in patients with SDHB-related tumors allow the use of CgA as a biomarker for further follow-up. Some SDHB-related tumors have been found to be biochemically silent owing to the lack of the enzyme tyrosine hydroxylase (60); perhaps CgA may prove to be helpful in their diagnosis.

### Imaging

Localization of neuroendocrine tumors should be pursued with imaging once biochemical evidence is established (see Figure 2). Renal ultrasound as the first modality of choice will miss small adrenal tumors and PGLs due to sensitivity and specificity of 89% (61) and 61% (62), respectively. Hence, CT or MRI of the abdomen and pelvis have been the imaging modality of choice in numerous pediatric cases (4, 17, 19, 20, 36, 63) given similar diagnostic sensitivities (90–100%) (50, 64). Specificities of both are around 70–80% (50, 64). MRI has better sensitivity than CT to locate extra-adrenal tumors and can evaluate the extent of invasion into the spinal canal and involvement of major vessels (4). Some advocate for MRI over CT scan in children given radiation exposure with CT (1).

As neither study is as specific in discerning a PCC/PGL from other abdominal pathology, functional imaging studies need to be pursued if one has a high index of suspicion for PCCs or PGLs, if a PGL is detected or if there are concerns for multifocal disease or malignancy. These include ^123^metaiodobenzylguanidine (^123^I-MIBG) scan, positron emission tomography (PET) with [^18^F] fluorodopamine (FDA), [^18^F] fluorodeoxyglucose (FDG), and [^18^F] fluorodihydroxyphenylalanine (F-DOPA). ^123^I-MIBG scan has been used in conjunction with CT/MRI in some studies to locate and rule out multifocal disease (4, 17, 19, 20, 64), offering 95–100% specificity in localizing PCCs/PGLs (50). The adult literature describes malignant PCCs/PGLs that lose the ability to accumulate this isotope, making a MIBG scan potentially negative in such cases (3). One needs to be mindful that tricyclic antidepressants, CCBs, and BBs (3) and over the counter decongestants interfere with tumor uptake of the iodinated isotope used in MIBG scans and their use should be discontinued prior to obtaining MIBG scans. [^18^F]-FDA PET has helped in defining and localizing tumor in a pediatric patient (65) and an adult patient with negative imaging but positive biochemical testing (66) and in the case of metastatic disease (3). In contrast, [^18^F]-FDG PET has been the recommended functional imaging technique to evaluate malignant and metastatic PCCs/PGLs, particularly in SDHB mutation carriers in adults (67). Recent advances in functional imaging of PCCs/PGLs have led to the use of radiolabeled DOTA peptides, such as [^68^Ga]-DOTATATE PET, which has high affinity for somatostatin receptor 2. Such receptors are known to be overexpressed in PCC/PGLs. Adult studies have demonstrated the superiority of [^68^Ga]-DOTATATE PET in localizing metastatic SDHB-associated PCCs/PGLs over the other functional imaging studies, excluding MIBG (68). [^68^Ga]-DOTATATE PET was also the most sensitive test in detecting HNPGLs (68, 69), especially SDHD tumors but inferior to F-DOPA PET/CT in detecting PCCs (69) in sporadic cases. Another study confirmed the high detection rate of PCCs/PGLs using [^68^Ga]-DOTATATE PET but noted that [^18^F]-FDG PET had higher uptake than the former in cases of mutations involving the pseudohypoxic cluster and a dedifferentiated tumor with loss of SSTR expression (70). In addition, CT with ^123^I-MIBG proved to have a lower lesion detection rate than [^68^Ga]-DOTATATE PET and [^18^F]-FDG PET in identifying PCCs and PGLs (70).

## Management

### Preoperative

Preoperative management of neuroendocrine tumors is crucial to prevent intraoperative complication of a hypertensive crisis. There has been a drastic decrease in perioperative complications from 45–69 to 3% with the use of alpha blockade (20, 63, 71, 72). Beta blockade is instituted following alpha blockade to offset reflex tachycardia from alpha-2 receptor antagonism and should never precede alpha blockade. This is due to the likelihood of causing a severe hypertensive crisis from unopposed alpha-receptor stimulation. Medications used preoperatively are discussed below with a goal BP reduction of <50 percentile for age and height. Dilated cardiomyopathy can develop from chronic catecholamine-induced hypertension, making an echocardiography valuable preoperatively (73, 74). One pediatric case report discusses the utility of pulmonary artery monitoring to assess fluid status in this setting, which has been described in adults (75).

Patients are asked to consume a high sodium diet of 6–10 g and fluid intake of at least 1.5 times maintenance a day once on an alpha blocker, to prevent hypotension from its vasodilatory properties (17, 36, 76). High fluid intake aids in expanding the contracted intravascular volume and reduces postoperative hypotension (77).

Patients are admitted to the hospital 24–36 h prior to surgery and given an alpha blocker, beta blocker, and in some cases a tyrosine hydroxylase inhibitor, the night before surgery (17, 36, 72, 78). Intravenous fluids of normal saline are given at 1.5 times maintenance to prevent hypotension prior to surgery (17, 36). Table 5 provides a list of medication regimens used in pediatric catecholamine-secreting tumors, with further information delineated as follows.

**Table 5:** Drugs used in preoperative blockade of pediatric catecholamine-secreting tumors.

Class of drug/drug name	Starting dose	Maintenance dose	Common side effects
Non-selective alpha blocker	0.2 mg/kg/day (max. 10 mg/dose)	Increase by 0.2 mg/kg/day every 4 days to goal 0.4–1.2 mg/kg/day ÷ every 6–8 h (max. 2–4 mg/kg/day)	Orthostatic hypotension
Phenoxybenzamine			Tachycardia
			Nasal congestion

Selective alpha-1 blocker	1–2 mg/day	Increase to 4–16 mg^a^, daily or ÷ 2 times daily	Orthostatic hypotension
Doxazosin			Dizziness

Non-selective beta blocker	1–2 mg/kg/day, ÷ 2–4 times daily	4 mg/kg/day, up to 640 mg/day, ÷ 2–4 times daily	Dizziness
Propranolol			Fatigue
			Asthma exacerbation

Selective beta-1 blocker	0.5–1 mg/kg/day, daily or ÷ 2 times daily	2 mg/kg/day, up to 100 mg/day, daily or ÷ 2 times daily	Edema
Atenolol			Dizziness
			Fatigue

Alpha and beta blocker	1–3 mg/kg/day, ÷ 2–3 times daily	10–12 mg/kg/day, up to 1,200 mg/day, ÷ 2–3 times daily	Dizziness
Labetalol			Fatigue
			Asthma exacerbation

Tyrosine hydroxylase inhibitor	20 mg/kg/day, ÷ every 6 h	Increase up to 60 mg/kg/day ÷ every 6 hrs	Orthostatic hypotension
Metyrosine	OR	OR	Diarrhea
	125 mg daily	Increase by 125 mg every 4–5 days to max. 2.5 g/day	Sedation
			Extra-pyramidal symptoms
			Crystalluria^b^

*^a^This study did not differentiate if the maximum dose of 16 mg was used in both pediatric and adult patients; however, review of pediatric dosing of doxazosin suggests a maximum dose of 4 mg/day. Titrating drug dosage to effect is recommended*.

*^b^Rare but potential side effect; with doses greater than 2 g/day, daily urine volume exceeding 2 L is recommended per manufacturer’s guidelines*.

#### Alpha Blockers

Phenoxybenzamine, a non-competitive alpha-1 and 2 adrenoreceptor antagonist, has been widely used since the 1950s to control hypertension preoperatively. Doxazosin or prazosin, competitive alpha-1 adrenoreceptor antagonists, are used by some adult centers since they do not cross the blood–brain barrier as does phenoxybenzamine, precluding central symptoms like headaches or nasal stuffiness (78). Reflex tachycardia is avoided with no alpha-2 adrenoreceptor blockade. The shorter duration of action of doxazosin or prazosin leads to little to no postural hypotension preoperatively, favoring their use in some adult case studies (78, 79).

Given the rarity of neuroendocrine tumors in pediatric and adult patients, there are no randomized controlled trials looking at the subtypes of medications. Numerous small pediatric case studies relate their experiences with preoperative management (4, 17, 36). Romero et al. delineated a stepwise approach to managing pediatric patients with neuroendocrine tumors (36). Phenoxybenzamine is given at a starting dose of 0.2 mg/kg/day once daily (max 10 mg/dose), increasing by 0.2 mg/kg/day every 4 days to reach a maintenance dose of 0.4–1.2 mg/kg/day divided every 6–8 h (max 2–4 mg/kg/day) 7–10 days prior to surgery (36).

Doxazosin has been used in children in a series of 50 patients, with a starting dose of 1–2 mg up to 16 mg, given daily or divided twice daily (18). Of note, this series also comprised adults, and the authors did not distinguish if the higher doses were used in pediatric patients.

The adult literature reviewed the efficacy of preoperative phenoxybenzamine, doxazosin, and prazosin in terms of having fewer hemodynamic fluctuations intraoperatively. A retrospective study showed no difference (80), a prospective study showed superiority of phenoxybenzamine over prazosin (76), and a retrospective study showed superiority of doxazosin over phenoxybenzamine (81).

#### Tyrosine Hydroxylase Inhibitor

Monotherapy with an alpha blocker has been found to cause hemodynamic instability intraoperatively during tumor manipulation. Metyrosine, a tyrosine hydroxylase inhibitor, prevents catecholamine synthesis and has been used in adults to prevent intraoperative BP fluctuations (79). The combination of phenoxybenzamine or prazosin and metyrosine in adults resulted in better BP control pre- and intraoperatively, with less need for pressure agents intraoperatively than when using phenoxybenzamine alone (79).

Ludwig et al. used metyrosine in 40% of their pediatric cases with 16% (one case) experiencing hemodynamic lability intraoperatively versus 50% (three cases) in those who did not receive this medication (*P* = 0.54) (17). They note their experience to be in agreement with the adult literature that metyrosine use is associated with a decreased need for intraoperative vasoactive medications, fluids, and decreased blood loss.

Perry et al. conducted a retrospective chart review of 25 adult patients with PCC, some of which received metyrosine in addition to phenoxybenzamine (72). Although there were no significant differences in BPs pre-, intra- and postoperatively, careful review of patients showed that those who received metyrosine had more severe disease and more stable BPs intraoperatively. Those who received the combination had less blood loss and less need for intraoperative fluids.

The pediatric literature does not have a consensus on the dosage of metyrosine. In addition to Ludwig et al. who used metyrosine, there are two case reports on children with catecholamine-secreting tumors who received metyrosine. The first dates back 24 years and used metyrosine in an 11 kg, 18-month-old child at a starting dose of 20 mg/kg/day divided every 6 h, titrated up to 60 mg/kg/day divided every 6 h (82). The other dates back 30 years and used metyrosine in a 12-year-old girl, weight ~30 kg. The starting dose of 125 mg daily was titrated up by 125 mg every 4–5 days to a maximum dose of 3.5 g daily (83). Metyrosine dose was reduced to 2.5 g after the patient experienced multiple oculogyric crises and received metyrosine for roughly 6 months, following which she underwent surgery. Most recent literature by Ludwig et al. (17) does not discuss the dosage or length of metyrosine used in pediatrics. In adults, dose of metyrosine starts at 250 mg every 6 h, titrated up by 250–500 mg daily to a maximum dose of 4 g/day, given 8–14 days prior to surgery (72, 79).

Based on the above data, pediatric dosing of metyrosine should start at either 20 mg/kg/day divided every 6 h, or 125 mg daily, whichever comes first based on the patient’s weight then titrated to maximum dose of 2.5 g daily over at least 8 days. The clinician should be mindful of the side effect profile of this medication (see Table 5), as well as signs of overdose to include persistent fatigue, anxiety, decreased salivation, dry mouth, diarrhea, and neuromuscular effects (tightening of the jaw, fine tremor of hands, and gross tremor of trunk), which may prompt reducing dose to prior step. This drug is available in 250 mg capsules, which can be opened and mixed to provide dilutions as appropriate for pediatric patients.

#### Beta Blockers

Beta blockers are generally used after alpha blockade has been instituted, to suppress reflex tachycardia. Standard pediatric dosing can be started at least 3 days prior to surgery (36) and should never be initiated prior to alpha blockade as this can lead to a hypertensive crisis. Tumors secreting primarily epinephrine (78) cause tachycardia and require a beta blocker.

### Intraoperative

There has been a shift in surgical expertise when resecting neuroendocrine tumors from laparotomy to laparoscopy in the adult (84, 85) and pediatric population (86, 87). Laparoscopic resection and adrenal cortical-sparing procedures are now the preferred approach, the latter being important in patients with bilateral adrenal disease, to preclude cortisol deficiency. Additional points of consideration in performing cortical-sparing surgery are the risk of disease recurrence, high malignancy rate and the likelihood of a patient to not require corticosteroids (88). Between 15 and 30% of the adrenal gland is needed to preserve function (89) and this, including the above points, should help in the decision tree to perform cortical-sparing surgery (88). Intraoperative hypertension is controlled with a variety of agents including sodium nitroprusside or esmolol (36). Magnesium sulfate, dexmedetomidine, or nicardipine have also been used (36, 75, 90). Care should be exercised when giving fluids intraoperatively if patients are hypotensive, to preclude cardiopulmonary complications in the case of catecholamine-induced cardiomyopathy (74, 77). Given the potential complications of resecting such tumors, an experienced anesthesiologist and endocrine surgeon are essential to the care of these patients.

### Postoperative

The use of phenoxybenzamine is associated with postoperative hypotension from sustained alpha blockade for the 24 h following surgery. This can be resistant to adrenergic arteriolar constrictors and instead requires intravascular fluids to maintain hemodynamic stability (78, 80). Postoperatively the sustained alpha blockade was not seen in patients receiving doxazosin as compared to phenoxybenzamine (78). Hypoglycemia can occur postoperatively due to rebound hyperinsulinism from a reduction of catecholamines and should be monitored (91).

### Treatment of Malignant PCCs and PGLs

Complete surgical resection is the curative therapy of PCCs and PGLs; in the instance of malignancy, certain therapies can offer disease control. ^131^I-MIBG is currently used for malignant tumors and relies on the uptake of MIBG by the norepinephrine transporter. If tumor uptake of this radioisotope is poor, other modalities to include peptide receptor radionuclide therapy with radiolabeled somatostatin analogs or ^111^In-pentetreotide scintigraphy (SRS) may be performed (92). Chemotherapy with cyclophosphamide, vincristine, and dacarbazine are also utilized with malignant disease in both pediatric and adult cases. The largest study to date has shown progressive disease in 52% (11 patients), while 4% (1 patient) had complete remission, 22% (5 patients) had partial response, and 22% (5 patients) had stable disease (93). Finally, external beam radiotherapy (EBRT) has been used to control metastatic PCCs and PGLs, with results of the largest retrospective study of 24 patients indicating symptomatic control in 81% of lesions and stable disease in 87% of lesions (94). A retrospective review of 17 patients with PGLs excluding HNPGLs were treated with EBRT with 76% of the patients achieving either local disease control or symptomatic relief (95). The retrospective nature of both studies does not allow for the determination of long-term disease progression or stability when using EBRT and hence prospective studies would be helpful.

## Survival, Recurrence, and Long-Term Follow-Up

A retrospective chart review of 30 pediatric patients, from 1975 to 2005 showed that those who were classified as having benign disease had a 100% survival rate as compared to those with malignant disease, who had 5-, 10-, and 15-year survival rates of 78, 62, and 31%, respectively (4). Genetic testing was not available during that time period except for five patients who tested positive for a RET mutation (three patients) or an SDHD or SDHB mutation (two patients). Authors from this study classified malignant disease as that with distant metastases, tumor unresectable due to local invasion of vital structures or tumor recurrence regionally or distally after initial resection and initial negative microscopic margins. Ludwig et al. reported on a 100% overall survival rate and 95% 5-year disease free survival, based on the lower malignancy rate of 7% in their patient population and their ability to achieve negative microscopic margins in all resections (17). Recent survival data from the EAPPR of the patients who were diagnosed in the pediatric age group show that 6% (8 patients) of those with hereditary disease (144 patients) died, with a follow-up mean range of 10–19 years (range 0–53 years) (11). Three of the patients had VHL, three had SDHB mutations, two had NF1, and one SDHA with the cause of death being metastases in seven and cardiac failure in one patient. All 33 patients with sporadic disease, followed for a mean of 10 years (range 1–45 years) were alive at subsequent follow-ups. The overall mean life expectancy of hereditary disease was 62 years. Life expectancy was greatly reduced with SDHB-associated disease, at 47 years, while patients with VHL had the lowest life expectancy at 27 years.

Malignant PCCs and PGLs are defined by the World Health Organization classification as the presence of metastases that does not include local invasion of a tumor (96). As such, a malignant tumor that has not yet metastasized may be classified as benign. Two classification systems exist to predict malignant potential of PCCs and PGLs. The PCC of the Adrenal Gland Scaled Score (PASS) (97) utilizes the histologic pattern, degree of cellularity, presence of necrosis, type of invasion and mitotic features to classify tumors while the Grading system for Adrenal Pheochromocytoma and Paraganglioma (GAPP) (98) additionally takes into account the Ki67 proliferation index of tumors as well as their biochemical profile. As user variability exists in the PASS system, it is not used in most centers. On the other hand, the GAPP system needs to be validated before being implemented into clinical practice. Given the lack of reliable markers to distinguish a benign lesion from a malignant one, lifelong follow-up of patients is required in the instance that metastases develop.

Long-term follow-up of these tumors is essential due to recurrence, which has been noted to occur anywhere between 1 and 14 years following initial presentation (11, 99, 100) in small pediatric case series. In contrast, data from the EAPPR showed that 38% of the pediatric registrants (*n* = 177) had recurrences after a mean time period of 25 years (11), with a reported recurrence rate of 12–38% (4, 11, 18, 100). The incidence of recurrent tumors increased with time, from 25% at 9 years to 50% at 31 years (11). The types of recurrent tumors were extra-adrenal (18%) and contralateral (13%) (95% CI 31–46) more so than ipsilateral (16%) ones (95% CI 12–28%) (11). Recurrences were significantly more common in patients with germline mutations than those with sporadic disease and tended to recur 10 years earlier, with a latency period of 23 versus 33 years, respectively (11). The mutations seen with these recurrent tumors were associated with VHL and SDHD mutations. Within these gene-specific mutations, SDHD mutations had a recurrent tumor after 18 years of latency versus 21 years for VHL mutations. HNPGLs recurred in 4% of pediatric patients and were caused by SDHD mutations at initial diagnosis and during recurrences. Seven percent of patients had a third recurrence, with a time interval of 1–20 years (mean 5 years) from second to third tumor; they all had germline mutations. The prevalence of malignancy was highest in SDHB mutation-positive individuals, with extra-adrenal and thoracic PGLs posing added risk for malignancy (11).

In a retrospective study of 263 patients with PCCs or PGLs, 125 were found to have metastatic disease, of which 32 patients presented before 20 years of age (42). Of those, 72% (23 patients) had a germline mutation in SDHB, 9.4% (3 patients) had an SDHD mutation, and 6.3% (2 patients) had a VHL mutation, with the absence of a known mutation in the remainder (4 patients). The study that established plasma methoxytyramine as a biomarker for metastatic PCCs and PGLs also recognized the association between extra-adrenal disease, tumor size >5 cm and SDHB mutation carriers associated with a high risk of malignancy (58).

Long-term follow-up on patients with hereditary PCCs and PGLs cannot be stressed enough given the lifelong risk of recurrence and metastatic disease. Laboratory testing with serum/urine metanephrines should be performed yearly and patients should undergo imaging studies intermittently and as clinically indicated based upon symptoms and/or positive laboratory testing (17) at follow-up visits. Smaller pediatric and adult case series recommend follow-up at 6 weeks and between 6 months and 1 year following initial surgery, then annually (17, 50).

The different characteristics of known mutations may change the follow-up frequency and surveillance emphasis. For example, SDHB mutations have high risk of metastasis (42), VHL and SDHD mutation carriers have high recurrence rates (11), and SDHA and TMEM127 have now been identified to confer added risks of malignancy. Recommendations of the EAPPR are to perform annual surveillance for the first 3 years after initial diagnosis of mutation carriers, this being the time frame where malignancy is apparent unless diagnosed at presentation, followed by lifelong follow-up. However, since malignancy can occur much later in life, constant and frequent follow-up is advisable in such patients.

## Conclusion

Pheochromocytoma and PGL, although rare, are potentially curable causes of secondary hypertension in pediatric patients. The identification of new gene mutations and the determination of recurrence and malignancy rates have allowed clinicians to acquire a better understanding of this disease process. All patients should be offered genetic testing given the high rate of inheritance of these tumors in pediatrics. All patients with genetic mutations should be followed throughout their lifetime given the risk of recurrence and malignancy. Those with SDHB gene mutations ought to be aggressively followed given the high risk of metastatic disease. Precise management of hypertension in such patients allows for safe pre-, intra-, and postoperative courses. It should be stressed that a multidisciplinary approach is needed in the treatment of patients with PCC or PGL. A team of experienced nephrologists, endocrinologists, genetic counselors, radiologists, endo-oncologists, oncologists, and surgeons will allow for optimal delivery of care to such patients and will also allow for the orchestration of follow-ups and careful attention to the recurrent and malignant potential of these tumors.

## Author Contributions

RB created the outline, performed a literature review, and wrote the manuscript; TB contributed expert knowledge and experience and edited the manuscript.

## Conflict of Interest Statement

The authors declare that the research was conducted in the absence of any commercial or financial relationships that could be construed as a potential conflict of interest.

## References

[1] Havekes B Romijn JA Eisenhofer G Adams K Pacak K. Update on pediatric pheochromocytoma. Pediatr Nephrol (2009) 24(5):943–50. 10.1007/s00467-008-0888-9 18566838

[2] Londe S Causes of hypertension in the young. Pediatr Clin North Am (1978) 25(1):55–65. 10.1016/S0031-3955(16)33532-5 628569

[3] Lenders JWM Eisenhofer G Mannelli M Pacak K Phaeochromocytoma. Lancet (2005) 366(9486):665–75. 10.1016/S0140-6736(05)67139-5 16112304

[4] Pham TH Moir C Thompson GB Zarroug AE Hamner CE Farley D Pheochromocytoma and paraganglioma in children: a review of medical and surgical management at a tertiary care center. Pediatrics (2006) 118(3):1109–17. 10.1542/peds.2005-2299 16951005

[5] Dobri GA Bravo E Hamrahian AH Pheochromocytoma: pitfalls in the biochemical evaluation. Expert Rev Endocrinol Metab (2014) 9(2):123–35. 10.1586/17446651.2014.887985 30743755

[6] Ciftci AO Tanyel FC Senocak ME Büyükpamukçu N. Pheochromocytoma in children. J Pediatr Surg (2001) 36(3):447–52. 10.1053/jpsu.2001.21612 11226993

[7] Neumann HP Bausch B McWhinney SR Bender BU Gimm O Franke G Germ-line mutations in nonsyndromic pheochromocytoma. N Engl J Med (2002) 346(19):1459–66. 10.1056/NEJMoa020152 12000816

[8] Barontini M Levin G Sanso G. Characteristics of pheochromocytoma in a 4- to 20-year-old population. Ann N Y Acad Sci (2006) 1073(1):30–7. 10.1196/annals.1353.003 17102069

[9] De Krijger RR Petri BJ Van Nederveen FH Korpershoek E De Herder WW De Muinck Keizer-Schrama SM Frequent genetic changes in childhood pheochromocytomas. Ann N Y Acad Sci (2006) 1073(1):166–76. 10.1196/annals.1353.017 17102083

[10] Vicha A Musil Z Pacak K. Genetics of pheochromocytoma and paraganglioma syndromes: new advances and future treatment options. Curr Opin Endocrinol Diabetes Obes (2013) 20(3):186–91. 10.1097/MED.0b013e32835fcc45 23481210 PMC4711348

[11] Bausch B Wellner U Bausch D Schiavi F Barontini M Sanso G Long-term prognosis of patients with pediatric pheochromocytoma. Endocr Relat Cancer (2014) 21(1):17–25. 10.1530/ERC-13-0415 24169644

[12] Adrogue HE Sinaiko AR. Prevalence of hypertension in junior high school-aged children: effect of new recommendations in the 1996 Updated Task Force Report. Am J Hypertens (2001) 14(5 Pt 1):412–4. 10.1016/S0895-7061(00)01277-2 11368459

[13] Sorof JM Lai D Turner J Poffenbarger T Portman RJ. Overweight, ethnicity, and the prevalence of hypertension in school-aged children. Pediatrics (2004) 113(3 Pt 1):475–82. 10.1542/peds.113.3.475 14993537

[14] Brady TM Feld LG Pediatric approach to hypertension. Semin Nephrol (2009) 29(4):379–88. 10.1016/j.semnephrol.2009.03.014 19615559

[15] Baracco R Kapur G Mattoo T Jain A Valentini R Ahmed M Prediction of primary vs secondary hypertension in children. J Clin Hypertens (Greenwich) (2012) 14(5):316–21. 10.1111/j.1751-7176.2012.00603.x 22533658 PMC8108809

[16] Kapur G Baracco R. Evaluation of hypertension in children. Curr Hypertens Rep (2013) 15(5):433–43. 10.1007/s11906-013-0371-2 23904150

[17] Ludwig AD Feig DI Brandt ML Hicks MJ Fitch ME Cass DL Recent advances in the diagnosis and treatment of pheochromocytoma in children. Am J Surg (2007) 194(6):792–6; discussion 796–7. 10.1016/j.amjsurg.2007.08.028 18005773

[18] Beltsevich DG Kuznetsov NS Kazaryan AM Lysenko MA. Pheochromocytoma surgery: epidemiologic peculiarities in children. World J Surg (2004) 28(6):592–6. 10.1007/s00268-004-7134-9 15366751

[19] Caty MG Coran AG Geagen M Thompson NW. Current diagnosis and treatment of pheochromocytoma in children. Experience with 22 consecutive tumors in 14 patients. Arch Surg (1990) 125(8):978–81. 10.1001/archsurg.1990.01410200036004 2378563

[20] Ein SH Pullerits J Creighton R Balfe JW. Pediatric pheochromocytoma. A 36-year review. Pediatr Surg Int (1997) 12(8):595–8. 10.1007/BF01371907 9354733

[21] Jain V Yadav J Satapathy AK Pheochromocytoma presenting as diabetes insipidus. Indian Pediatr (2013) 50(11):1056–7. 24382904

[22] Eisenhofer G Goldstein DS Sullivan P Csako G Brouwers FM Lai EW Biochemical and clinical manifestations of dopamine-producing paragangliomas: utility of plasma methoxytyramine. J Clin Endocrinol Metab (2005) 90(4):2068–75. 10.1210/jc.2004-2025 15644397

[23] Fishbein L Merrill S Fraker DL Cohen DL Nathanson KL. Inherited mutations in pheochromocytoma and paraganglioma: why all patients should be offered genetic testing. Ann Surg Oncol (2013) 20(5):1444–50. 10.1245/s10434-013-2942-5 23512077 PMC4291281

[24] Gill AJ Chou A Vilain R Clarkson A Lui M Jin R Immunohistochemistry for SDHB divides gastrointestinal stromal tumors (GISTs) into 2 distinct types. Am J Surg Pathol (2010) 34(5):636–44. 10.1097/PAS.0b013e3181d6150d 20305538

[25] Carney JA. Gastric stromal sarcoma, pulmonary chondroma, and extra-adrenal paraganglioma (Carney Triad): natural history, adrenocortical component, and possible familial occurrence. Mayo Clin Proc (1999) 74(6):543–52. 10.4065/74.6.543 10377927

[26] Welander J Söderkvist P Gimm O. Genetics and clinical characteristics of hereditary pheochromocytomas and paragangliomas. Endocr Relat Cancer (2011) 18(6):R253–76. 10.1530/ERC-11-0170 22041710

[27] Stratakis CA Carney JA The triad of paragangliomas, gastric stromal tumours and pulmonary chondromas (Carney triad), and the dyad of paragangliomas and gastric stromal sarcomas (Carney–Stratakis syndrome): molecular genetics and clinical implications. J Intern Med (2009) 266(1):43–52. 10.1111/j.1365-2796.2009.02110.x 19522824 PMC3129547

[28] Vaughan P Pabla L Hobin D Barron DJ Parikh D. Cardiac paraganglioma and gastrointestinal stromal tumor: a pediatric case of Carney-Stratakis syndrome. Ann Thorac Surg (2011) 92(5):1877–8. 10.1016/j.athoracsur.2011.03.123 22051283

[29] Pacak K Jochmanova I Prodanov T Yang C Merino MJ Fojo T New syndrome of paraganglioma and somatostatinoma associated with polycythemia. J Clin Oncol (2013) 31(13):1690–8. 10.1200/JCO.2012.47.1912 23509317 PMC3807138

[30] Därr R Nambuba J Del Rivero J Janssen I Merino M Todorovic M Novel insights into the polycythemia-paraganglioma-somatostatinoma syndrome. Endocr Relat Cancer (2016) 23(12):899–908. 10.1530/ERC-16-0231 27679736 PMC5096964

[31] Pacak K Chew EY Pappo AS Yang C Lorenzo FR Wilson MW Ocular manifestations of hypoxia-inducible factor-2α paraganglioma-somatostatinoma-polycythemia syndrome. Ophthalmology (2014) 121(11):2291–3. 10.1016/j.ophtha.2014.06.019 25109928 PMC4253312

[32] Qin Y Yao L King EE Buddavarapu K Lenci RE Chocron ES Germline mutations in TMEM127 confer susceptibility to pheochromocytoma. Nat Genet (2010) 42(3):229–33. 10.1038/ng.533 20154675 PMC2998199

[33] Comino-Méndez I Gracia-Aznárez FJ Schiavi F Landa I Leandro-García LJ Letón R Exome sequencing identifies MAX mutations as a cause of hereditary pheochromocytoma. Nat Genet (2011) 43(7):663–7. 10.1038/ng.861 21685915

[34] Kirmani S Young WF Hereditary paraganglioma-pheochromocytoma syndromes. In: Pagon RA Adam MP Ardinger HH, editors. GeneReviews^®^ [Internet]. Seattle, WA: University of Washington, Seattle (2008). 1993–2017.

[35] Bausch B Schiavi F Ni Y Welander J Patocs A Ngeow J Clinical characterization of the pheochromocytoma and paraganglioma susceptibility genes SDHA, TMEM127, MAX, and SDHAF2 for gene-informed prevention. JAMA Oncol (2017) E1–9. 10.1001/jamaoncol.2017.0223 PMC5824290 28384794

[36] Romero M Kapur G Baracco R Valentini RP Mattoo TK Jain A. Treatment of hypertension in children with catecholamine-secreting tumors: a systematic approach. J Clin Hypertens (Greenwich) (2015) 17(9):720–5. 10.1111/jch.12571 26010736 PMC8031986

[37] Toyoda H Hirayama J Sugimoto Y Uchida K Ohishi K Hirayama M Polycythemia and paraganglioma with a novel somatic HIF2A mutation in a male. Pediatrics (2014) 133(6):e1787–91. 10.1542/peds.2013-2419 24819565

[38] Neumann HPH Eng C The approach to the patient with paraganglioma. J Clin Endocrinol Metab (2009) 94(8):2677–83. 10.1210/jc.2009-0496 19657044 PMC2730863

[39] Waguespack SG Rich T Grubbs E Ying AK Perrier ND Ayala-Ramirez M A current review of the etiology, diagnosis, and treatment of pediatric pheochromocytoma and paraganglioma. J Clin Endocrinol Metab (2010) 95(5):2023–37. 10.1210/jc.2009-2830 20215394

[40] Pamporaki C Hamplova B Peitzsch M Prejbisz A Beuschlein F Timmers HJLM Characteristics of pediatric vs adult pheochromocytomas and paragangliomas. J Clin Endocrinol Metab (2017) 102(4):1122–32. 10.1210/jc.2016-3829 28324046 PMC5460722

[41] Reddy VS O’Neill JA Jr Holcomb GW III Neblett WW III Pietsch JB Morgan WM III Twenty-five-year surgical experience with pheochromocytoma in children. Am Surg (2000) 66(12):1085–91; discussion 1092. 11149577

[42] King KS Prodanov T Kantorovich V Fojo T Hewitt JK Zacharin M Metastatic pheochromocytoma/paraganglioma related to primary tumor development in childhood or adolescence: significant link to SDHB mutations. J Clin Oncol (2011) 29(31):4137–42. 10.1200/JCO.2011.34.6353 21969497 PMC3208535

[43] van Hulsteijn LT Dekkers OM Hes FJ Smit JW Corssmit EP. Risk of malignant paraganglioma in SDHB-mutation and SDHD-mutation carriers: a systematic review and meta-analysis. J Med Genet (2012) 49(12):768–76. 10.1136/jmedgenet-2012-101192 23099648

[44] Gill AJ Benn DE Chou A Clarkson A Muljono A Meyer-Rochow GY Immunohistochemistry for SDHB triages genetic testing of SDHB, SDHC, and SDHD in paraganglioma-pheochromocytoma syndromes. Hum Pathol (2010) 41(6):805–14. 10.1016/j.humpath.2009.12.005 20236688

[45] Lenders JW Duh QY Eisenhofer G Gimenez-Roqueplo AP Grebe SK Murad MH Pheochromocytoma and paraganglioma: an endocrine society clinical practice guideline. J Clin Endocrinol Metab (2014) 99(6):1915–42. 10.1210/jc.2014-1498 24893135

[46] Lenders JW Pacak K Walther MM Linehan WM Mannelli M Friberg P Biochemical diagnosis of pheochromocytoma: which test is best? JAMA (2002) 287(11):1427–34. 10.1001/jama.287.11.1427 11903030

[47] Eisenhofer G Keiser H Friberg P Mezey E Huynh TT Hiremagalur B Plasma metanephrines are markers of pheochromocytoma produced by catechol-O-methyltransferase within tumors. J Clin Endocrinol Metab (1998) 83(6):2175–85. 10.1210/jcem.83.6.4870 9626157

[48] Weise M Merke DP Pacak K Walther MM Eisenhofer G. Utility of plasma free metanephrines for detecting childhood pheochromocytoma. J Clin Endocrinol Metab (2002) 87(5):1955–60. 10.1210/jcem.87.5.8446 11994324

[49] Eisenhofer G Goldstein DS Walther MM Friberg P Lenders JW Keiser HR Biochemical diagnosis of pheochromocytoma: how to distinguish true- from false-positive test results. J Clin Endocrinol Metab (2003) 88(6):2656–66. 10.1210/jc.2002-030005 12788870

[50] Pacak K Linehan WM Eisenhofer G Walther MM Goldstein DS Recent advances in genetics, diagnosis, localization, and treatment of pheochromocytoma. Ann Intern Med (2001) 134(4):315–29. 10.7326/0003-4819-134-4-200102200-00016 11182843

[51] Eisenhofer G Lattke P Herberg M Siegert G Qin N Därr R Reference intervals for plasma free metanephrines with an age adjustment for normetanephrine for optimized laboratory testing of phaeochromocytoma. Ann Clin Biochem (2013) 50(Pt 1):62–9. 10.1258/acb.2012.012066 23065528 PMC4714582

[52] Deutschbein T Unger N Jaeger A Broecker-Preuss M Mann K Petersenn S. Influence of various confounding variables and storage conditions on metanephrine and normetanephrine levels in plasma. Clin Endocrinol (2010) 73(2):153–60. 10.1111/j.1365-2265.2009.03761.x 20039892

[53] Hoy LJ Emery M Wedzicha JA Davison AG Chew SL Monson JP Obstructive sleep apnea presenting as pseudopheochromocytoma: a case report. J Clin Endocrinol Metab (2004) 89(5):2033–8. 10.1210/jc.2003-031348 15126517

[54] de Jong WH Eisenhofer G Post WJ Muskiet FA de Vries EG Kema IP. Dietary influences on plasma and urinary metanephrines: implications for diagnosis of catecholamine-producing tumors. J Clin Endocrinol Metab (2009) 94(8):2841–9. 10.1210/jc.2009-0303 19567530

[55] Kaditis AG Alexopoulos EI Damani E Hatzi F Chaidas K Kostopoulou T Urine levels of catecholamines in Greek children with obstructive sleep-disordered breathing. Pediatr Pulmonol (2009) 44(1):38–45. 10.1002/ppul.20916 19085921

[56] Eisenhofer G Huysmans F Pacak K Walther MM Sweep FC Lenders JW. Plasma metanephrines in renal failure. Kidney Int (2005) 67(2):668–77. 10.1111/j.1523-1755.2005.67123.x 15673315

[57] Eisenhofer G Lenders JW Timmers H Mannelli M Grebe SK Hofbauer LC Measurements of plasma methoxytyramine, normetanephrine, and metanephrine as discriminators of different hereditary forms of pheochromocytoma. Clin Chem (2011) 57(3):411–20. 10.1373/clinchem.2010.153320 21262951 PMC3164998

[58] Eisenhofer G Lenders JW Siegert G Bornstein SR Friberg P Milosevic D Plasma methoxytyramine: a novel biomarker of metastatic pheochromocytoma and paraganglioma in relation to established risk factors of tumour size, location and SDHB mutation status. Eur J Cancer (2012) 48(11):1739–49. 10.1016/j.ejca.2011.07.016 22036874 PMC3372624

[59] Zuber S Wesley R Prodanov T Eisenhofer G Pacak K Kantorovich V. Clinical utility of chromogranin A in SDHx-related paragangliomas. Eur J Clin Invest (2014) 44(4):365–71. 10.1111/eci.12245 24467715

[60] Timmers HJ Pacak K Huynh TT Abu-Asab M Tsokos M Merino MJ Biochemically silent abdominal paragangliomas in patients with mutations in the succinate dehydrogenase subunit B gene. J Clin Endocrinol Metab (2008) 93(12):4826–32. 10.1210/jc.2008-1093 18840642 PMC2626451

[61] Lucon AM Pereira MA Mendonça BB Halpern A Wajchenbeg BL Arap S. Pheochromocytoma: study of 50 cases. J Urol (1997) 157(4):1208–12. 10.1097/00005392-199704000-00005 9120903

[62] Abrams HL Siegelman SS Adams DF Sanders R Finberg HJ Hessel SJ Computed tomography versus ultrasound of the adrenal gland: a prospective study. Radiology (1982) 143(1):121–8. 10.1148/radiology.143.1.7063713 7063713

[63] Goldstein RE O’Neill JA Jr Holcomb GW III Morgan WM III Neblett WW III Oates JA Clinical experience over 48 years with pheochromocytoma. Ann Surg (1999) 229(6):755–64; discussion 764–6. 10.1097/00000658-199906000-00001 10363888 PMC1420821

[64] Ilias I Pacak K Current approaches and recommended algorithm for the diagnostic localization of pheochromocytoma. J Clin Endocrinol Metab (2004) 89(2):479–91. 10.1210/jc.2003-031091 14764749

[65] Levine DS Metzger DL Nadel HR Oviedo A Adam MJ Skarsgard E. Novel use of F-DOPA PET/CT imaging in a child with paraganglioma/pheochromocytoma syndrome. Pediatr Radiol (2011) 41(10):1321–5. 10.1007/s00247-011-2109-0 21567141

[66] Pacak K Eisenhofer G Carrasquillo JA Chen CC Li ST Goldstein DS. 6-[18F]fluorodopamine positron emission tomographic (PET) scanning for diagnostic localization of pheochromocytoma. Hypertension (2001) 38(1):6–8. 10.1161/01.HYP.38.1.6 11463751

[67] Timmers HJ Kozupa A Chen CC Carrasquillo JA Ling A Eisenhofer G Superiority of fluorodeoxyglucose positron emission tomography to other functional imaging techniques in the evaluation of metastatic SDHB-associated pheochromocytoma and paraganglioma. J Clin Oncol (2007) 25(16):2262–9. 10.1200/JCO.2006.09.6297 17538171

[68] Janssen I Blanchet EM Adams K Chen CC Millo CM Herscovitch P Superiority of [68Ga]-DOTATATE PET/CT to other functional imaging modalities in the localization of SDHB-associated metastatic pheochromocytoma and paraganglioma. Clin Cancer Res (2015) 21(17):3888–95. 10.1158/1078-0432.CCR-14-2751 25873086 PMC4558308

[69] Archier A Varoquaux A Garrigue P Montava M Guerin C Gabriel S Prospective comparison of 68Ga-DOTATATE and 18F-FDOPA PET/CT in patients with various pheochromocytomas and paragangliomas with emphasis on sporadic cases. Eur J Nucl Med Mol Imaging (2016) 43(7):1248–57. 10.1007/s00259-015-3268-2 26637204

[70] Chang CA Pattison DA Tothill RW Kong G Akhurst TJ Hicks RJ 68Ga-DOTATATE and 18F-FDG PET/CT in paraganglioma and pheochromocytoma: utility, patterns and heterogeneity. Cancer Imaging (2016) 16(1):22 10.1186/s40644-016-0084-2 27535829 PMC4989291

[71] Fishbein L Orlowski R Cohen D. Pheochromocytoma/paraganglioma: review of perioperative management of blood pressure and update on genetic mutations associated with pheochromocytoma. J Clin Hypertens (Greenwich) (2013) 15(6):428–34. 10.1111/jch.12084 23730992 PMC4581847

[72] Perry RR Keiser HR Norton JA Wall RT Robertson CN Travis W Surgical management of pheochromocytoma with the use of metyrosine. Ann Surg (1990) 212(5):621–8. 10.1097/00000658-199011000-00010 1978640 PMC1358191

[73] Kinney MAO Narr BJ Warner MA Perioperative management of pheochromocytoma. J Cardiothorac Vasc Anesth (2002) 16(3):359–69. 10.1053/jcan.2002.124150 12073213

[74] Turner MC Lieberman E DeQuattro V The perioperative management of pheochromocytoma in children. Clin Pediatr (Phila) (1992) 31(10):583–9. 10.1177/000992289203101002 1395364

[75] Kalra Y Agarwal HS Smith AH Perioperative management of pheochromocytoma and catecholamine-induced dilated cardiomyopathy in a pediatric patient. Pediatr Cardiol (2013) 34(8):2013–6. 10.1007/s00246-012-0564-5 23132179

[76] Agrawal R Mishra SK Bhatia E Mishra A Chand G Agarwal G Prospective study to compare peri-operative hemodynamic alterations following preparation for pheochromocytoma surgery by phenoxybenzamine or prazosin. World J Surg (2014) 38(3):716–23. 10.1007/s00268-013-2325-x 24233658

[77] Bravo EL Tagle R. Pheochromocytoma: state-of-the-art and future prospects. Endocr Rev (2003) 24(4):539–53. 10.1210/er.2002-0013 12920154

[78] Prys-Roberts C Farndon JR. Efficacy and safety of doxazosin for perioperative management of patients with pheochromocytoma. World J Surg (2002) 26(8):1037–42. 10.1007/s00268-002-6667-z 12192533

[79] Steinsapir J Carr AA Prisant LM Bransome ED Jr. Metyrosine and pheochromocytoma. Arch Intern Med (1997) 157(8):901–6. 10.1001/archinte.157.8.901 9129550

[80] Kocak S Aydintug S Canakci N. Alpha blockade in preoperative preparation of patients with pheochromocytomas. Int Surg (2002) 87(3):191–4. 12403097

[81] Zhu Y He HC Su TW Wu YX Wang WQ Zhao JP Selective alpha1-adrenoceptor antagonist (controlled release tablets) in preoperative management of pheochromocytoma. Endocrine (2010) 38(2):254–9. 10.1007/s12020-010-9381-x 21046486

[82] Caballero R Pirmohamed R Wright JA Use of alpha-methyl-tyrosine for refractory hypertension in a child with neuroblastoma. Crit Care Med (1992) 20(7):1060–2. 10.1097/00003246-199207000-00026 1352196

[83] Imperato-McGinley J Gautier T Ehlers K Zullo MA Goldstein DS Vaughan ED Jr Reversibility of catecholamine-induced dilated cardiomyopathy in a child with a pheochromocytoma. N Engl J Med (1987) 316(13):793–7. 10.1056/NEJM198703263161307 2881206

[84] Brunt LM Lairmore TC Doherty GM Quasebarth MA DeBenedetti M Moley JF. Adrenalectomy for familial pheochromocytoma in the laparoscopic era. Ann Surg (2002) 235(5):713–20; discussion 720–1. 10.1097/00000658-200205000-00014 11981218 PMC1422498

[85] Kazaryan AM Kuznetsov NS Shulutko AM Beltsevich DG Edwin B. Evaluation of endoscopic and traditional open approaches to pheochromocytoma. Surg Endosc (2004) 18(6):937–41. 10.1007/s00464-003-9199-1 15108109

[86] Castilho LN Castillo OA Dénes FT Mitre AI Arap S. Laparoscopic adrenal surgery in children. J Urol (2002) 168(1):221–4. 10.1097/00005392-200207000-00080 12050547

[87] Miller KA Albanese C Harrison M Farmer D Ostlie DJ Gittes G Experience with laparoscopic adrenalectomy in pediatric patients. J Pediatr Surg (2002) 37(7):979–82; discussion 979–82. 10.1053/jpsu.2002.33822 12077753

[88] Yip L Surgical management of hereditary pheochromocytoma1. J Am Coll Surg (2004) 198(4):525–34. 10.1016/j.jamcollsurg.2003.12.001 15051000

[89] Brauckhoff M Stock K Stock S Lorenz K Sekulla C Brauckhoff K Limitations of intraoperative adrenal remnant volume measurement in patients undergoing subtotal adrenalectomy. World J Surg (2008) 32(5):863–72. 10.1007/s00268-007-9402-y 18224482

[90] Pacak K Eisenhofer G Ahlman H Bornstein SR Gimenez-Roqueplo AP Grossman AB Pheochromocytoma: recommendations for clinical practice from the First International Symposium. October 2005. Nat Clin Pract Endocrinol Metab (2007) 3(2):92–102. 10.1038/ncpendmet0396 17237836

[91] Hack HA. The perioperative management of children with phaeochromocytoma. Paediatr Anaesth (2000) 10(5):463–76. 10.1046/j.1460-9592.2000.00504.x 11012949

[92] van Hulsteijn LT Niemeijer ND Dekkers OM Corssmit EP 131I-MIBG therapy for malignant paraganglioma and phaeochromocytoma: systematic review and meta-analysis. Clin Endocrinol (2014) 80(4):487–501. 10.1111/cen.12341 24118038

[93] Asai S Katabami T Tsuiki M Tanaka Y Naruse M. Controlling tumor progression with cyclophosphamide, vincristine, and dacarbazine treatment improves survival in patients with metastatic and unresectable malignant pheochromocytomas/paragangliomas. Horm Cancer (2017) 8(2):108–18. 10.1007/s12672-017-0284-7 28108930 PMC10355862

[94] Vogel J Atanacio AS Prodanov T Turkbey BI Adams K Martucci V External beam radiation therapy in treatment of malignant pheochromocytoma and paraganglioma. Front Oncol (2014) 4:166. 10.3389/fonc.2014.00166 25019060 PMC4073229

[95] Fishbein L Bonner L Torigian DA Nathanson KL Cohen DL Pryma D External beam radiation therapy (EBRT) for patients with malignant pheochromocytoma and non-head and neck paraganglioma: combination with (131)I-MIBG. Horm Metab Res (2012) 44(5):405–10. 10.1055/s-0032-1308992 22566196 PMC4357844

[96] DeLellis R Lloyd R Heitz P Eng C Pathology and Genetics of Tumours of Endocrine Organs (2004). Lyon: IARC Press.

[97] Thompson LD. Pheochromocytoma of the adrenal gland scaled score (PASS) to separate benign from malignant neoplasms: a clinicopathologic and immunophenotypic study of 100 cases. Am J Surg Pathol (2002) 26(5):551–66. 10.1097/00000478-200205000-00002 11979086

[98] Kimura N Takayanagi R Takizawa N Itagaki E Katabami T Kakoi N Pathological grading for predicting metastasis in phaeochromocytoma and paraganglioma. Endocr Relat Cancer (2014) 21(3):405–14. 10.1530/ERC-13-0494 24521857

[99] Kim HY Lee HS Jung SE Lee SC Park KW Kim WK Experience with surgical excision in childhood pheochromocytoma. J Korean Med Sci (2004) 19(3):401–6. 10.3346/jkms.2004.19.3.401 15201507 PMC2816842

[100] Ein SH Shandling B Wesson D Filler Rm. Recurrent pheochromocytomas in children. J Pediatr Surg (1990) 25(10):1063–5. 10.1016/0022-3468(90)90219-Y 2262859

